# Leveraging paired germline and somatic analysis to improve the classification of *DDX41* variants

**DOI:** 10.1111/bjh.70411

**Published:** 2026-03-23

**Authors:** Andrew George, Elisabeth Rolf, Monika Domeradzka, Sara Ribeiro, Brittany Mills, April Hallett, Suzanne Macmahon, Paula Proszek, Ridwan Shaikh, Lina Yuan, Michael Hubank, Mikel Valganon Petrizan, Terri P. McVeigh, Jamshid S. Khorashad

**Affiliations:** ^1^ Clinical Genomics, Centre for Molecular Pathology The Royal Marsden NHS Foundation Trust Sutton UK; ^2^ Division of Genetics and Epidemiology The Institute of Cancer Research Sutton UK; ^3^ Cancer Genetics Unit The Royal Marsden NHS Foundation Trust Sutton UK; ^4^ Division of Cancer Biology The Institute of Cancer Research Sutton UK

**Keywords:** ACMG, CanVIG, *DDX41*, MDS

## Abstract

Constitutional pathogenic variants in *DDX41* predispose to myelodysplasia and acute myeloid leukaemia. Acquisition of subsequent somatic hits in the second allele is frequent, with notable recurrent variants at key hotspots. Sequencing of Deoxyribonucleic acid from blood/marrow of 239 patients with suspected/confirmed haematological malignancies at a single centre within a 4‐year period identified 136 unique *DDX41* variants. Among those with co‐occurring somatic and likely/confirmed germline variants, 54.8% of likely/confirmed germline variants were pathogenic when classified according to current Cancer Variant Interpretation Group UK (CanVIG‐UK) guidelines (incorporating American College of Medical Genetics [ACMG] criteria), while 45.2% were deemed variants of uncertain significance (VUS). Classification of variants as uncertain poses challenges, as it then calls into question the underlying aetiology of the malignancy, as well as the significance of any subsequent somatic *DDX41* variants. As carrier relatives of suspected deleterious *DDX41* variants will not usually be considered as donors for bone marrow transplantation, classification of variants of likely germline origin will have immediate treatment implications. Current ACMG and CanVIG‐UK guidelines do not permit co‐occurrence with recurrent somatic driver variants as evidence favouring pathogenicity, despite this being a convincing finding. This study proposes modifying certain rules as a basis for developing *DDX41*‐specific guidance, as it will significantly impact decisions surrounding bone marrow transplantation.

## INTRODUCTION

Detection of relevant driver variants is increasingly used for diagnosis, risk classification and treatment selection in haematological malignancies.^1^ Some patients carry inherited genetic risk factors; when likely germline variants are detected, their constitutional origin should be confirmed for clinical management.^2^ In leukaemia patients with inherited risk factors, this influences treatment decisions, particularly bone marrow transplantation, as relatives carrying the same genetic risk factor should not serve as donors.^2^, ^3^, ^4^ Additionally, identifying heritable risk factors has implications for patients' relatives.

Germline testing identifies the causes of heritable malignancies to inform the clinical management of patients and at‐risk relatives. Misattribution may lead to incorrect diagnosis/prognosis, false reassurance for non‐carrier relatives or catastrophic decisions such as terminating ‘affected’ embryos. Interpreting germline variants is challenging, with high thresholds for clinical actionability in proband management and in pre‐symptomatic, prenatal or preimplantation testing. Conversely, somatic testing identifies likely drivers to refine diagnosis, provide prognosis and inform therapy. Short turnaround times necessitate brief interpretation, leading to lower thresholds for classifying somatic variants as drivers than for germline pathogenic classification. However, when somatic variants occur at frequencies suggesting germline origin, hereditary implications should be considered, requiring interpretation according to germline classification guidance.^2^ In NHS diagnostic laboratories throughout the United Kingdom, variants in cancer susceptibility genes are typically interpreted and classified according to the Cancer Variant Interpretation Group UK (CanVIG‐UK) Consensus Specification, which is guided by the variant interpretation framework established by the Association for Clinical Genomic Science (ACGS), based on the American College of Medical Genetics (ACMG).^5^ In addition to the general guidelines, several gene‐specific guidelines have also been developed, including CanVIG‐UK gene‐specific guidance and gene‐specific guidance from ClinGen Sequence Variant Interpretation (SVI).^6^ For certain genes with associated unique and predictable characteristics, the application of generic guidelines in the absence of gene‐specific guidance may inadvertently lead to a large proportion of variants being classified as ‘uncertain’, even where suspicion is high based on features not currently considered as part of the existing variant interpretation framework. Such features include a second well‐established oncogenic somatic hit in the normal allele, the absence of other drivers or other molecular characteristics.

Presymptomatic genetic testing for at‐risk family members is typically offered only for alterations classified as (likely) pathogenic. It is recommended that patients carrying suspected inherited alterations in genes associated with blood cancers be referred to clinical geneticists to discuss further management. In rare instances, it may be necessary to offer testing for uncertain alterations to facilitate decisions regarding bone marrow transplantation.^2^ Reporting variants of likely germline origin as ‘uncertain’ when detected in the somatic context is challenging, as it raises questions about the diagnosis, prognosis and management of the proband. There may also be inconsistencies in reporting between patients—the same variant may be reported as a likely driver when occurring as a somatic event if germline variant interpretation guidance is not applied. Efforts are ongoing by the UK Somatic Variant Interpretation Group (UK SVIG) and others to align somatic and germline variant interpretation guidelines, but risks persist while these are being awaited.^7^

DDX41 is a multifunctional protein involved in Ribonucleic acid (RNA) splicing, R‐loop resolution, ribosome biogenesis and innate immune sensing.^8^ Pathogenic *DDX41* mutations reduce or abolish these protein functions, leading to replication stress, increased double‐strand Deoxyribonucleic acid (DNA) break formation and transcription alterations associated with inflammatory response.^9^, ^10^, ^11^, ^12^ Pathogenic germline variants in *DDX41* are the most common heritable cause of myelodysplasia (MDS) and acute myeloid leukaemia (AML). Predisposition to malignancy is inherited as a dominant trait, with a penetrance of nearly 50%.^13^, ^14^, ^15^, ^16^ Characteristically, the phenotype presents late in adulthood, with a typical age of onset in the sixth decade or later.^9^ Germline *DDX41* mutations are predominantly loss‐of‐function variants (63–68%), including frameshift, nonsense and splice‐site alterations, concentrated in the N terminus upstream of the DEAD box domain, with p.M1I and p.D140fs being the most common in Caucasian populations.^15^, ^17^ These heterozygous mutations result in haploinsufficiency of this essential DEAD‐box helicase, leading to loss of tumour suppressor function, primarily affecting RNA splicing and processing pathways.^18^ Notably, complete DDX41 inactivation appears to be cell lethal, which explains why germline frameshift mutations are never accompanied by somatic deletions and why patients typically acquire a second‐hit hypomorphic mutation (most commonly R525H) rather than a complete loss‐of‐function variant, as some residual DDX41 activity appears necessary for haematopoietic stem cell survival.^19^, ^20^

There is a relative lack of data regarding the functional impact of most missense variants in this gene. Furthermore, the relatively late onset of the associated phenotype, combined with incomplete penetrance, complicates the application of evidence related to population variant frequency or case–control analyses. Second somatic hits in the other allele are frequent (~80%), with recurrent oncogenic variants at key hotspots, but, at present, the generic ACGS guidance does not account for such co‐occurrence as an evidence point favouring pathogenicity. For many cancer predisposition genes, observations indicate that the inherited germline variant may increase susceptibility to the somatic second hit.^21^, ^22^ At present, the frequent occurrence of variants in the somatic context is not evidence that can be considered for germline variant interpretation when generic germline classification guidelines are applied, although information from somatic variation has been adopted for tumour protein p53 (*TP53*)‐specific germline variant interpretation.^23^, ^24^ Given the high frequency of certain *DDX41* variants in the somatic context, the omission of these data represents a missed opportunity to better characterise these variants when detected during germline testing.

*DDX41*‐specific criteria for germline variant classification represent a critical gap in the current literature. Our single‐institution retrospective survey addresses this gap by systematically analysing *DDX41* variants in a well‐characterised patient cohort, providing the clinical evidence base needed to develop *DDX41*‐specific pathogenicity criteria. Although limited to a single institution, this focused approach allows for detailed clinical correlation and standardised data collection, which are essential first steps towards establishing variant classification guidelines that can be validated in larger multicentre studies.

## MATERIALS AND METHODS

### Case selection

The Clinical Genomics department at the Royal Marsden Hospital (RMH) analysed routine diagnostic haemato‐oncology samples using UKAS15189‐accredited RMH200 Haemonc panel between October 2020 and June 2024 and re‐assessed them for the presence of *DDX41* variants. The DNA for the sequencing was extracted from either peripheral blood or bone marrow aspirate. The use of data for evaluating and improving the interpretation, reporting and management of variants generated by the RMH Haemonc panel was reviewed and approved by the RMH Committee for Clinical Review (CCR) (Ref No: SE1430).

Please refer to the supplementary materials for the information about the RMH200 Haemonc panel, bioinformatics, germline copy number analysis, germline validation and variant classification and interpretation.

## RESULTS

**FIGURE 1:**
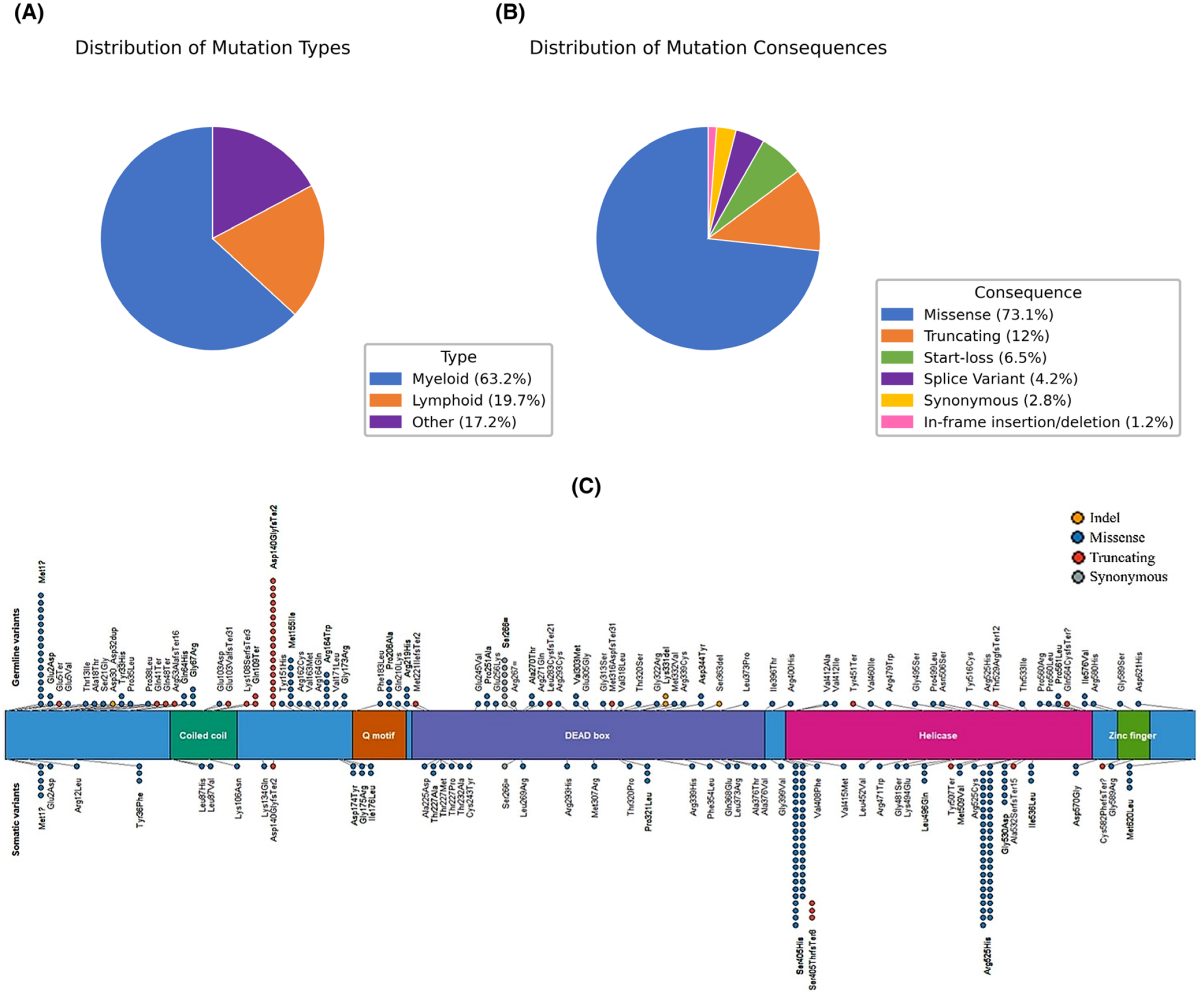
(A) Pie chart showing the breakdown of disease phenotypes among samples in which at least one *DDX41* variant was identified. (B) Pie chart depicting the functional consequence classification of all detected *DDX41* variants. (C) The figure displays the distribution of variants along the full‐length DDX41 protein. Germline variants are shown in the upper track, while somatic variants identified in tumour samples are shown in the lower track. Each dot represents an individual variant, with the vertical position indicating its frequency in the samples. Variants are colour‐coded by functional consequence. Key functional domains of the DEAD‐box helicase family are highlighted in coloured bars, including the coiled coil, Q motif, DEAD box, helicase domain and zinc finger motif.

Two hundred and thirty‐nine patients with suspected/confirmed haematological malignancies were identified as having missense, frameshift, indels or splice‐site variants in *DDX41*. Clinical indications were distributed as follows: 151 (63.2%) myeloid, 47 (19.7%) lymphoid and 41 (17.1%) non‐specific/not specified/other (Figure 1A). Analysis of *DDX41* variants identified 136 unique variants, including 55 somatic and 85 potentially germline (Figure 1B, 1C and Table S1). Four variants were seen at various VAFs consistent with either germline or somatic origin: c.415_418dup, c.798G>A, c.6G>T and c.1574G>A (Table S1). Of the (likely) germline variants, p.(Asp140GlyfsTer2) and p.(Met1?) (c.3G>A) were the most common. 87/239 (36.4%) patients had only somatic variants, and 90/239 (37.7%) had only potentially germline variants. Sixty‐two patients had at least two *DDX41* variants, with at least one being a potential germline (Table 1); in four cases, more than one co‐occurring somatic *DDX41* variant was observed (Table S1).

**TABLE 1:** Patients with co‐occurring potentially germline and somatic *DDX41* variants.

Patient #	Referral diagnosis	Age range	HGVS variant	Germline variant pathogenicity according to CanVIG/ACMG	Variant 2	VAF	Previously reported
RMH1 169	Leucopenia	64	c.435‐2_435‐1delinsCA	P	P321L	0.21	31, 32
RMH1 036	AML	76	c.936‐1G>C	L/P	R525H	0.07	^a^
RMH1 020	AML	79	c.3G>A p.(Met1?)	L/P	R525H	0.03	^a^
RMH1 122	Cytopenia	78	c.3G>A p.(Met1?)	L/P	R525H	0.03	^a^
RMH1 098	AML	79	c.3G>A p.(Met1?)	L/P	R525H	0.03	^a^
RMH1 075	Pancytopenia	82	c.3G>A p.(Met1?)	L/P	G530D	0.03	32, 33
RMH1 107	Pancytopenia	69	c.3G>A p.(Met1?)	L/P	R525H	0.05	^a^
RMH1 072	MDS	72	c.3G>A p.(Met1?)	L/P	R525H	0.06	^a^
RMH1 104	Myeloid Leukaemia	62	c.3G>A p.(Met1?)	L/P	L496Q	0.06	N
RMH1 023	Pancytopenia	60	c.3G>A p.(Met1?)	L/P	G530D	0.10	32, 33
RMH1 147	AML	88	c.3G>A p.(Met1?)	L/P	L496Q	0.13	^a^
RMH1045	AML	72	c.3G>A p.(Met1?)	L/P	G530D	0.16	32, 33
RMH1 031	MDS	69	c.13G>T p.(Glu5Ter)^b^	VUS	R525H	0.35	^a^
RMH1 207	Pancytopenia	70	c.113C>T p.(Pro38Leu)	VUS	T227A	0.27	N
RMH1 117	Myeloid leukaemia	70	c.121C>T p.(Gln41Ter)	L/P	R525H	0.06	^a^
RMH1 216	Unspecified acute leukaemia	56	c.142C>T p.(Gln48Ter)	L/P	R525H	0.007	^a^
RMH1 025	AML	80	c.308_309del p.(Glu103ValfsTer31)	L/P	R525H	0.41	^a^
RMH1 137	MDS	87	c.323del p.(Lys108SerfsTer3)	L/P	L87H	0.01	N
RMH1 143	MDS	78	c.325C>T p.(Gln109Ter)^c^	VUS	R525H	0.21	^a^
RMH1 130	MDS	86	c.325C>T p.(Gln109Ter)^c^	VUS	F354L	0.38	N
RMH1 054	MDS	67	c.415_418dup p.(Asp140GlyfsTer2)	L/P	R525H	0.02	^a^
RMH1 057	MDS	74	c.415_418dup p.(Asp140GlyfsTer2)	L/P	R525H	0.02	^a^
RMH1 004	Pancytopenia	66	c.415_418dup p.(Asp140GlyfsTer2)	L/P	R525H	0.02	^a^
RMH1 126	MDS	75	c.415_418dup p.(Asp140GlyfsTer2)	L/P	R525H	0.04	^a^
RMH1 133	AML	88	c.415_418dup p.(Asp140GlyfsTer2)	L/P	A225D	0.05	^32^
RMH1 134	AML	83	c.415_418dup p.(Asp140GlyfsTer2)	L/P	R525H	0.07	^a^
RMH1 093	AML	69	c.415_418dup p.(Asp140GlyfsTer2)	L/P	R525H	0.07	^a^
RMH1 072	AML	71	c.415_418dup p.(Asp140GlyfsTer2)	L/P	R525H	0.07	^a^
RMH1 047	AML	63	c.415_418dup p.(Asp140GlyfsTer2)	L/P	R525H	0.08	^a^
RMH1 079	Aplastic Anaemia	58	c.415_418dup p.(Asp140GlyfsTer2)	L/P	R525H	0.10	^a^
RMH1 191	AML	64	c.415_418dup p.(Asp140GlyfsTer2)	L/P	R525H	0.12	^a^
RMH1 033	Pancytopenia	59	c.415_418dup p.(Asp140GlyfsTer2)	L/P	R525H	0.26	^a^
RMH1 172	MDS	72	c.415_418dup p.(Asp140GlyfsTer2)	L/P	P321L	0.32	31, 32
RMH1 028	AML	76	c.415_418dup p.(Asp140GlyfsTer2)	L/P	R525H	0.36	^a^
RMH1 015	MDS	82	c.415_418dup p.(Asp140GlyfsTer2)	L/P	D570G	0.05	N
					P321L	0.01	31, 32
RMH1 141	MDS	74	c.465G>A p.(Met155Ile)	VUS	R471W	0.16	N
					T227M	0.12	N
					D570G	0.009	N
RMH1 166	MDS	69	c.465G>A p.(Met155Ile)	VUS	R525H	0.35	^a^
RMH1 211	Unspecified acute leukaemia	67	c.490C>T p.(Arg164Trp)	VUS	S405H	0.43	N
RMH1 203	Pancytopenia	75	c.517G>A p.(Gly173Arg)	VUS	R525H	0.06	^a^
RMH1 081	MDS	61	c.517G>A p.(Gly173Arg)	VUS	R525H	0.12	^a^
RMH1 106	MDS	70	c.549C>A p.(Phe183Leu)	VUS	G530D	0.26	32, 33
RMH1 213	CLL	82	c.656G>A p.(Arg219His)	VUS	L87V	0.42	N
RMH1 200	MDS	77	c.663del p.(Met221IlefsTer2)	L/P	R525H	0.03	^a^
RMH1 125	MDS	66	c.766G>A p.(Glu256Lys)	VUS	R525H	0.33	N
RMH1 056	MDS	73	c.808G>A p.(Ala270Thr)	VUS	R525H	0.30	^a^
RMH1 171	MDS	70	c.808G>A p.(Ala270Thr)	VUS	R525H	0.32	^a^
RMH1 077	Pancytopenia	95	c.847del p.(Leu283CysfsTer21)	P	R525H	0.01	^a^
RMH1 111	Neutropenia	67	c.952G>T p.(Val318Leu)	VUS	T227A	0.18	N
RMH1 124	Polycythaemia	75	c.964G>A p.(Gly322Arg)	VUS	R525H	0.07	^a^
RMH1016	Pancytopenia	60	c.992_994del p.(Lys331del)	VUS	G530D	0.11	32, 33
RMH1 042	AML	94	c.992_994del p.(Ile576Val)	VUS	G530D	0.11	32, 33
					T232A	0.04	N
RMH1 219	MDS	74	c.1015C>T p.(Arg339Cys)	VUS	A376V	0.28	N
RMH1 144	Pancytopenia	78	c.1030G>T p.(Asp344Tyr)	VUS	G589R	0.03	N
RMH1 082	MDS	75	c.1030G>T p.(Asp344Tyr)	VUS	R525H	0.16	^a^
RMH1 105	Neutropenia	79	c.1088_1090del p.(Ser363del) c.97T>C p.(Tyr33His)	VUS L/B	L496Q	0.10	N
RMH1 176	AML	77	c.465G>A p.(Ile396Thr)	VUS	G399V	0.37	N
RMH1 145	MDS	79	c.490C>T p.(Gly495Ser)	VUS	Q368E	0.21	N
RMH1 189	Pancytopenia	76	c.517G>A p.(Pro499Leu)	VUS	V415M	0.006	N
RMH1 092	MDS	59	c.517G>A p.(Tyr516Cys)	VUS	R525H	0.01	^a^
RMH1 078	Pancytopenia	61	c.549C>A p.(Thr529ArgfsTer12)	P	R525H	0.13	^a^
RMH1 160	Pancytopenia	48	c.656G>A p.(Thr533Ile)	VUS	A376T	0.20	N
RMH1 076	AML	67	c.663del^d^ p.(Pro560Leu)	VUS			^15^
RMH1 146	Pancytopenia	84	c.1690_1693del p.(Gln564CysfsTer?)	P	R525H	0.12	^a^
RMH1024	MPN	15	c.1726GA>G p.(Lys331del)	VUS	S405H	0.04	N
					K134Q	0.03	N
					c.1732+2T>A	0.05	N

*Note*: The somatic variants are presented as a one‐letter code format in this table.

Abbreviations: L/B, likely benign; L/P, likely pathogenic; MPN, Myeloproliferative Neoplasms; N, not reported before this study; P, pathogenic; VUS, variant of uncertain significance.

^a^ The most common somatic *DDX41* variant in this study and the literature.

^b^ A stop codon is generated within the first 100 bp of the first exon. NMD not predicted as possible reinitiation at Met127. The function of the region lost is unknown, but it encompasses more than 10% of the protein.

^c^ ≥2 concordant evidence items are required for a classification of likely pathogenic/pathogenic. Currently, there is insufficient evidence.

^d^ The VAF for this variant was 95%, indicating more than one event.

### Germline variants co‐occurring with somatic variants

#### Sequence variants

Among the 62 patients harbouring both potentially germline *DDX41* variants and co‐occurring somatic variants, a distinct pattern of somatic alterations emerged. The R525H somatic variant was predominant, occurring in 35 patients (56.5%), followed by the G530D variant in six patients (9.7%). This distribution highlights the recurrent nature of specific somatic *DDX41* alterations in patients with underlying germline susceptibility.

Classification of germline variants according to CanVIG‐UK guidelines revealed that 34 of the 62 patients (54.8%) carried variants classified as pathogenic or likely pathogenic, while the remaining 28 patients (45.2%) harboured a VUS (Table 1). The co‐occurrence of a germline *DDX41* VUS with an established oncogenic somatic *DDX41* variant substantially increases the likelihood that the germline variant contributes to the patient's phenotype, particularly when no additional genetic alterations are identified in either germline or somatic contexts.^25^ Analysis of the germline VUS revealed several recurrent alterations, with A270T (Patients RMH1 056 and RMH1 171), D344Y (Patients RMH1 082 and RMH1 144) and M155I (Patients RMH1 141, RMH1 116, RMH1 149, RMH1 158, RMH1 166, RMH1 167 and RMH1 199) each identified in multiple patients (Table S1). Notably, only the M155I variant had been previously documented in the gnomAD v4.1 database, with a low population frequency of 0.0002301. The rarity of these variants in population databases, combined with their recurrent appearance in our cohort, supports their potential pathogenicity. The co‐occurrence patterns between germline VUS and (likely) pathogenic somatic variants provided additional evidence for pathogenicity. Carriers of the (likely) germline A270T variant (Patients RMH1 056 and RMH1 171) consistently exhibited the recurrent somatic R525H alteration. Similarly, one D344Y carrier (Patient RMH1 082) also acquired the R525H somatic variant, while the other (Patient RMH1 144) acquired the G589R somatic variant. Among M155I carriers, one patient (Patient RMH1 166) presented with the R525H somatic variant, while another (Patient RMH1 141) exhibited multiple somatic alterations, including the recurrent T227M variant alongside R471W and R570G missense variants (Table S1). These patterns suggest that specific germline variants may predispose to the acquisition of particular somatic alterations, potentially reflecting underlying mechanistic relationships in *DDX41*‐related pathogenesis.

#### Copy number variants

**FIGURE 2:**
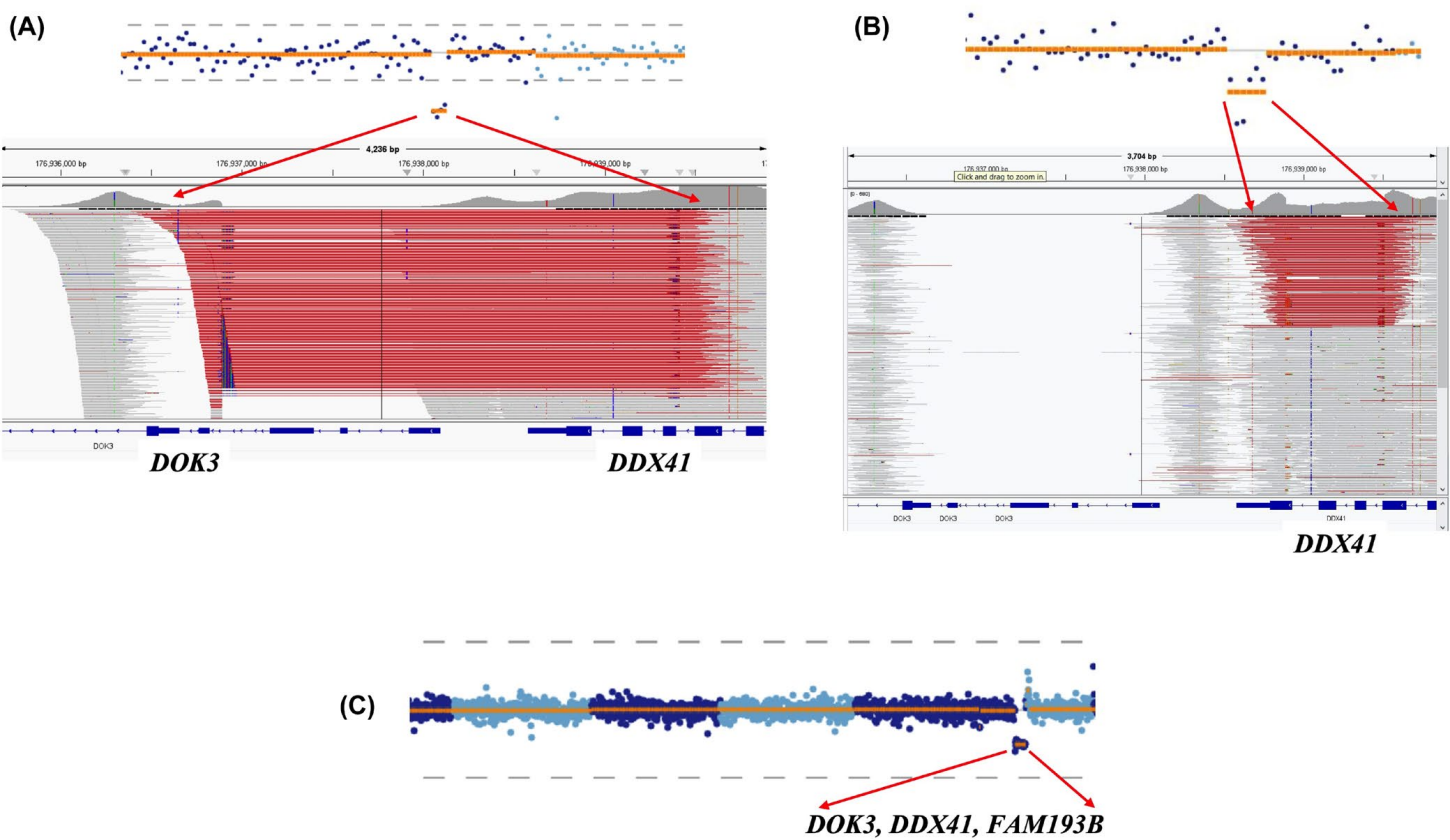
Spectrum of germline *DDX41* copy number alterations. (A) Partial copy number loss affecting the terminal four exons of *DDX41* and extending beyond the gene boundary (Patient RMH1 049) and (B) focal deletion encompassing exons 13 (partial), 14 and 15 of the *DDX41* gene are shown as observed on the CNV plot (Top) and Integrative Genomic Viewer (IGV) (below) (Patient RMH1 041). Each dot in the CNV plots represents a probe that captures a specific genomic sequence. Probes that change together relative to the reference set are grouped into segments, as indicated by the orange line. In the IGV, the deletion is represented as red lines. (C) Large‐scale deletion spanning the entire *DDX41* locus and adjacent upstream and downstream genes (Patient RMH1 231).

Somatic variants affecting the R525 residue were identified in 45 patients, comprising 44 cases with R525H and one case with R525C (Table S1). This represents the most frequently altered residue in our cohort, emphasising its critical role in *DDX41*‐related pathogenesis. As outlined above, co‐occurring germline SNVs (VAF 40%–60%) were identified in 35 patients. The remaining 10 patients did not exhibit apparent germline *DDX41* SNVs but showed alternative genetic patterns. Three of these patients (RMH1 049, RMH1 231, RMH1 041) carried germline complete or partial deletions of *DDX41*, confirmed by copy number analysis (Figure 2). These findings indicate that structural variants may serve as the germline ‘first hit’ in *DDX41*‐related disorders. Among the six patients with uninformative germline sequence and copy number analyses, three distinct patterns emerged: (1) *Dual somatic variants*: Three patients (RMH1 003, RMH1 087, RMH1 165) exhibited the somatic R525H variant co‐occurring with a different somatic *DDX41* variant. Notably, both variants demonstrated similar VAFs, suggesting they likely originated within the same malignant clone rather than representing independent subclonal events. (2) *Alternative pathway involvement*: One patient (Patient RMH1 120), despite lacking additional *DDX41* alterations, exhibited multi‐hit pathogenic *TP53* variants and a complex chromosomal pattern, indicating an alternative mechanism of oncogenesis. (3) *Isolated R525 variants*: Two patients (Patient RMH1 034, RMH1 115) demonstrated only the single somatic R525 variant, with no additional detectable *DDX41* alterations in either germline or somatic contexts, despite comprehensive sequence and copy number analyses (Table S1). These findings highlight the importance of comprehensive genomic analysis to fully characterise the *DDX41* mutational pattern in these patients.

#### *DDX41* variants in patients with lymphoid malignancy

*DDX41* germline variants were not limited to patients with myeloid malignancies; they were also found in lymphoid malignancies (Table 2). The p(Met1?) variant, one of the most prevalent likely pathogenic germline *DDX41* variants, was identified in two patients with chronic lymphocytic leukaemia (CLL), while another CLL patient had two *DDX41* potentially germline variants (R219H and L87V) (Tables 1 and 2).

**TABLE 2:** The observed potentially germline *DDX41* variant in lymphoid malignancies.

Patient #	Age range	Disease	Germline variant	VAF	Population frequency
RMH1 196	58	B‐ALL	p.(Tyr33His)	0.4097	0.000065
RMH1 073	34	T‐ALL	p.(Glu2Asp)	0.5225	0.0000732
RMH1 167	71	B cell Non‐Hodgkin Lymphoma (NHL)	p.(Met155Ile)	0.4801	0.000223
RMH1 090	68	B cell NHL	p.(Arg267=)	0.4779	0.0000161
RMH1 099	69	B cell NHL	c.434 + 1G>A	0.5125	
RMH1 083	59	CLL	p.(Arg219His)	0.6175	0.0000318
RMH1 178	78	CLL	p.(Arg219His)	0.4915	0.0000318
RMH1 213	82	CLL	p.(Arg219His)	0.5197	0.0000318
			p.(Leu87Val)	0.427	
RMH1 094	72	CLL	p.(Met1?)	0.4826	8.15E‐05
RMH1 095	93	CLL	p.(Met1?)	0.4731	8.15E‐05
RMH1 013	37	CLL	p.(Met332Val)	0.4911	0.000004
RMH1 035	50	CLL	p.(Pro561Leu)	0.5019	
RMH1 037	69	Plasma cell myeloma	p.(Asp621His)	0.9333	
RMH1 103	73	Plasma cell myeloma	p.(Gly67Arg)	0.6639	0.000008
RMH1 112	97	Plasma cell myeloma	p.(Pro206Ala)	0.6392	0.0000477
RMH1 021	76	T‐cell lymphoma	p.(Gln210Lys)	0.4747	

### Comparing RMH cohort and UK biobank

To contextualise our findings within a broader population framework, we compared the frequency and classification of *DDX41* variants identified in our cohort with those reported in the recent UK Biobank *DDX41* analysis.^26^ Several variants identified as somatic events in our cohort were reported as likely germline variants in the UK Biobank study. These discrepancies included: *G175R variant*: Detected in two patients (RMH1 006, RMH1 212) in our cohort as somatic events with low VAFs of 1.3% and 1.1%, suggesting acquired rather than inherited alterations. *P321L variant*: Identified as a second somatic hit in three carriers of pathogenic or likely pathogenic germline variants, occurring at VAFs of 1.7%, 21.8% and 33% (RMH1 015, RMH1 168, RMH1 172 respectively). The variable VAF range suggests different timing of acquisition during disease progression. Additional variants that were detected exclusively as somatic events included R293H (2.0% VAF) (RMH1 051), R339H (8.4% VAF) (RMH1 192) and R471W (16.7% VAF) (RMH1 141) (Table S1).

In the biobank study, strict variant classification using ACMG criteria was not applied. Rather, germline variants were deemed pathogenic if they were stop‐gain, frameshift or essential splice variants or if previously reported to co‐occur with *DDX41* somatic variants in MDS/AML samples or pre‐MDS/AML samples in their UKB cohort. When germline variant classification guidance is applied, 28 of these germline variants are classified as VUS.^26^ Ten of these reclassified VUS were also identified in our cohort: E256K, G173R, G313S, I396T, K331del, L373P, Q210K, R339C, S363del and Y516C. Importantly, somatic analysis of carriers harbouring seven of these variants (E256K, G173R, I396T, K331del, R339C, S363del, Y516C) revealed the presence of second, somatic *DDX41* variants (Table 1). This pattern of germline VUS co‐occurring with somatic variants provides compelling evidence for the pathogenic significance of these germline alterations, supporting a two‐hit model of *DDX41*‐related disease pathogenesis.

## DISCUSSION

Robust classification of *DDX41* variants is crucial for accurate diagnosis as well as providing valuable information to guide prognosis and, importantly, facilitate donor selection when bone marrow transplantation is a consideration. Multiple lines of evidence must be used to support the classification of variants and minimise misclassification. The co‐occurrence of a germline variant with a known oncogenic somatic variant, as an isolated line of evidence, is insufficient to enable classification of a variant as (likely) pathogenic, given that somatic drivers may occur as causal events, even in individuals without an underlying heritable predisposition. Including data on somatic variant frequency, co‐occurrence with known oncogenic somatic variants, clinical phenotype, population frequency and, when available, case–control data may provide enough evidence to more definitively classify a novel *DDX41* variant that might otherwise remain a VUS.

While the standard ACMG/CanVIG‐UK guidelines are essential, they do not account for the unique biology of *DDX41*‐associated malignancies. In our cohort, the high proportion of VUS (45.2%), despite concurrent somatic mutations, underscores the need to supplement current classification frameworks, which are not specifically tailored to *DDX41*. While isolated somatic drivers can occur in individuals without heritable predisposition, the recurrent association of specific germline variants with somatic mutations in our study suggests a functional relationship whereby germline variants contribute to malignant transformation. In our cohort, 77% (36/47) of pathogenic/likely pathogenic germline variants had co‐occurring somatic variants, with R525H (25/36) predominating, followed by G530D (3/36), P321L (3/36), L496Q (2/36) and isolated cases of L87H, A225D and R471W. This non‐random distribution—particularly the strong association with recurrent somatic hotspots like R525H—indicates that germline variants predispose to malignant transformation through acquisition of specific second somatic hits. This pattern is fundamentally different from germline variants that occur coincidentally with unrelated somatic drivers.

The presence of specific paired germline–somatic variants is not addressed by the current ACMG/ACGS frameworks and should be incorporated into pathogenicity assessment rather than dismissed as confounding evidence. We propose that, in the context of *DDX41*, evidence of a second somatic *DDX41* variant should provide explicit evidence for germline pathogenicity. Drawing on precedent from *TP53* VCEP guidelines,^27^ where paired germline–somatic mutations are recognised as strong evidence for tumour suppressor pathogenicity, we recommend incorporating this criterion into *DDX41*‐specific guidance. The specific strength of evidence remains under discussion—previous studies have suggested PM3 (allelic data) at a moderate level or PP4 (phenotype specificity) at a very strong level—but clarifying how to weight paired germline–somatic data should be a priority for future *DDX41* classification frameworks. For example, a germline *DDX41* variant that recurrently pairs with somatic R525H (a known oncogenic hotspot in AML) demonstrates functional relevance through its strong association with malignant transformation. This association alone should elevate classification beyond what the ACMG criteria applied in isolation would allow.

Accurate application of PS4 requires comparative frequency data between cases and controls. For *DDX41*, case–control data are limited and complicated by age‐related and incomplete penetrance, which significantly restricted our ability to assign PS4 at stronger evidence levels in this study. This represents a major gap: many germline variants with suggestive evidence for pathogenicity cannot be definitively classified due to insufficient population‐level data. We recommend establishing a centralised national database of *DDX41*‐positive cases, leveraging the UK Genomic Medicine Service to pool variants across diagnostic laboratories. Integration with existing resources such as the CanVIG database and alternative ‘quasi case–control’ approaches—using disease prevalence calculations and large control datasets such as UK Biobank—could provide robust data for PS4 application. This infrastructure would not only improve real‐time variant classification but also enable ongoing refinement of *DDX41*‐specific guidance as additional cases accrue.

When germline variants do not demonstrate a clear association with somatic hotspots or somatic co‐drivers, multiple other lines of evidence become essential for robust classification. In our cohort, the absence of clear mutational hotspots or regional enrichment prevented application of PM1 criteria; however, integration of population frequency, in silico predictions, functional domain location, clinical phenotype and case–control data (where available) alongside somatic co‐occurrence data provided sufficient evidence to move several variants from VUS to likely pathogenic classification. This multi‐evidence approach acknowledges that somatic‐germline concordance is one of several overlapping lines of evidence—none individually sufficient but collectively compelling.

A major limitation in current *DDX41* variant classification is the lack of functional characterisation for many reported variants. *DDX41* has relatively few published functional studies, creating challenges for interpreting missense and non‐canonical splice‐site variants, which are often classified as VUS. Within ACMG frameworks, functional evidence is weighted by the number of both pathogenic and benign controls. In practice, studies often utilise only a wild‐type control as the benign comparator, limiting the strength of evidence. Future standardised assays incorporating appropriate pathogenic and benign control variants are needed to provide higher confidence functional data, enabling stronger application of PS3/BS3 in *DDX41*‐specific guidance.

Application of population frequency data is contentious, given that the rarity of a variant in population databases is not necessarily strongly indicative of pathogenicity, and conversely, relative commonality should not necessarily preclude pathogenic classification of a variant associated with incomplete penetrance. Ethnicity‐specific differences in the frequency and spectrum of germline *DDX41* variants complicate the application of rules based on population frequency. Age‐ and gender‐related adjustments to *DDX41*‐specific PM2 thresholds that account for factors influencing penetrance should be considered in future *DDX41* guidance.

An important technical consideration is the necessity of comprehensive variant detection capabilities. In our cohort, three patients harboured germline partial or complete *DDX41* deletions that would be missed without copy number variation analysis. We recommend that copy number loss of the *DDX41* gene be systematically investigated in patients with isolated somatic hotspot R525 variants; however, a larger study is required to provide stronger evidence for this recommendation.

The main reason for the increased detection of potential *DDX41* germline variants in recent years is the adoption of Next‐generation sequencing as a diagnostic tool for managing AML/MDS patients. The NHS Test Directory includes a list of cancer‐specific genes for screening, among which is the *DDX41* gene for AML or MDS patients. This has led to increased analysis of this gene, resulting in the discovery of unique variants. Incomplete penetrance and later onset of the phenotype mean that family histories are often unhelpful in predicting an underlying pathogenic germline *DDX41* variant; therefore, most germline variants are discovered inadvertently through somatic testing rather than through initial germline testing. These ascertainment patterns necessitate robust variant interpretation frameworks that do not rely on classical family history evidence.

The predominant phenotype in carriers of pathogenic *DDX41* variants is predisposition to myeloid neoplasms, but other features have also been reported in affected individuals, including multiple myeloma, non‐Hodgkin and Hodgkin lymphoma, acute and chronic lymphocytic leukaemia and autoimmune disorders, as well as potential susceptibility to solid organ cancers,^28^, ^29^, ^30^ although further data are required to confirm these associations. Recognition of this broader phenotypic spectrum is important for variant interpretation, particularly when applying PP4 criteria based on phenotype–genotype correlation.

In the United Kingdom, concerted efforts through CanVIG‐UK have enabled the rapid development of gene‐specific variant interpretation guidance and modifications to other expert guidelines, allowing for practicable and standardised approaches suitable for NHS laboratories. We recommend modification of rules as presented in Table 3 in the context of *DDX41*‐specific guidance. Leveraging this existing network to facilitate the development of a national database with prospectively maintained phenotypic and genotypic information from individuals with *DDX41*‐associated disease or carriers of germline *DDX41* variants will be valuable to inform *DDX41‐*specific variant interpretation guidance. This will reduce the number of germline variants of uncertain significance and improve the clinical actionability of the *DDX41* findings.

**TABLE 3:** The Royal Marsden Hospital recommended modification to *DDX41* germline variant classification.

Recommendation 1	Integration of the two‐hit mechanism of pathogenicity into variant interpretation. *DDX41*‐associated malignancies typically arise through a two‐hit mechanism, with an initial germline pathogenic variant and an acquired somatic ‘second hit’. Current ACMG/ACGS frameworks do not explicitly account for this mechanism in variant interpretation. We propose that, in the context of *DDX41*, the presence of a second, somatic *DDX41* variant should provide additional evidence for the pathogenicity of the germline variant. The appropriate strength of evidence remains under discussion: prior studies have suggested applying this criterion as PM3 (allelic data) at a moderate level^15^ or PP4 (phenotype specificity) at very strong level.^25^ Clarifying how best to incorporate paired germline–somatic data should be a priority for future *DDX41*‐specific guidance.
Recommendation 2	Database development to support PS4 application. The accurate application of PS4 requires knowledge of how frequently a variant occurs in affected individuals compared to controls, which enables the calculation of the odds ratio. For *DDX41*, case–control data are limited, which restricted the ability to assign PS4 at stronger levels in this study. Alternative ‘quasi case–control’ approaches, such as those based on disease prevalence and large control datasets (e.g. UK Biobank), may provide partial solutions.^34^ However, the preferred approach would be to establish a centralised database of *DDX41*‐positive cases, ideally leveraging the UK Genomic Medicine Service to pool variants across diagnostic laboratories and integrating these with existing resources such as the CanVIG database.
Recommendation 3	Functional assays to support missense variant interpretation. A major limitation in current *DDX41* variant classification is the lack of functional characterisation for many reported variants. In contrast to well‐studied cancer predisposition genes, *DDX41* has relatively few published functional studies. This creates challenges for the interpretation of missense and non‐canonical splice site variants, which often remain classified as VUS. Functional evidence in ACMG/ACGS frameworks is weighted by the number of both pathogenic and benign controls included in an assay. In practice, studies often utilise a wild‐type control as the only benign control, limiting the strength of evidence that can be applied. In our study, no functional evidence was used for this reason. Future standardised assays that incorporate appropriate control variants are needed to provide higher confidence functional data, enabling stronger application of PS3/BS3 in *DDX41*‐specific guidelines.
Recommendation 4	Modification of PM2 thresholds. In generic guidance, PM2 can be applied at a supporting level if a variant is absent from gnomAD or present at a frequency ≤0.002%. However, ethnicity‐specific differences in the frequency and spectrum of germline *DDX41* variants have been reported,^35^ which complicates the application of this rule. For example, in our dataset, the truncating variant c.142C>T p.(Gln48Ter) was observed at an allele frequency of 0.01% in the African/African American subpopulation, preventing PM2 assignment despite its likely pathogenicity. Gene‐specific adjustment of PM2 thresholds that account for population substructure should be considered in future *DDX41* guidance.

## AUTHOR CONTRIBUTIONS

AG, ER, SR, BM, AH and JSK generated the data. AG, ER, SR, MD and JSK analysed the data. PP, RS, LY and MH designed the assay and the bioinformatic pipeline. LY, MH and MVP reviewed data generation. ER, TPM and JSK wrote the manuscript. SM, MH and MVP reviewed the manuscript.

## CONFLICT OF INTEREST STATEMENT

The authors declare no conflicts of interest.

## Supporting information


Data S1.

## Data Availability

The data that support the findings of this study are available from the corresponding author upon reasonable request.
