# Supplementary material for: Leveraging paired germline and somatic analysis to improve the classification of DDX41 variants
Source: Br J Haematol. 2026 Mar 23;208(5):1584–95. doi: 10.1111/bjh.70411 (PMC13176513; doi:10.1111/bjh.70411)
Supplement: Supplementary file 1 — Data S1. [file BJH-208-1584-s001.docx]

### Targeted next‐generation sequencing

The HaemOnc NGS panel was designed to sequence the genes recommended by the NHS Test Directory for haematological malignancies. NGS libraries were prepared from 25-400 ng of DNA using the NGS STAR Hamilton Automated Liquid Handler and the Kapa HyperPlus kit (Roche) along with IDT UDI 8bp adapters (Integrated DNA Technologies, Coralville, USA), following the manufacturer’s protocol, which included dual-SPRI size selection of the libraries (300-500 bp). To optimise enrichment and reduce off-target capture, pooled, multiplexed, amplified pre-capture libraries (up to 24 libraries per capture) were hybridised overnight using 1 µg of total DNA to a custom panel, Haemonc v1 (Roche, USA). The hybridised DNA was cleaned up and PCR amplified, and the products were purified using AMPure XP beads (Beckman Coulter, Danvers, MA, USA). The captures were then quantified using the Qubit dsDNA High Sensitivity Assay Kit with the Qubit 3.0 fluorometer (Invitrogen, Carlsbad, CA, USA), as well as the High Sensitivity D1000 TapeStation (Agilent, Santa Clara, USA). Sequencing was performed on a NovaSeq6000 (Illumina, San Diego, CA, USA) with 150 bp paired-end reads.

Bioinformatics

Analysis was conducted using the Illumina DRAGEN Bio-IT on-premises platform via a bespoke analytical pipeline developed at the Centre for Molecular Pathology, The Royal Marsden Hospital, to facilitate the identification of Small Nucleotide Variants (SNVs) and Copy Number Variants (CNVs). Raw data were demultiplexed into fastq files with DRAGEN BCLconvert. The analysis was performed on the DRAGEN Bio-IT platform v3.10 based on the hg19 human reference genome, with variants annotated using PCGR (https://github.com/sigven/pcgr): a wrapper for Variant Effect Predictor (VEP) v105, which encompasses several variant databases, including, but not limited to, VEP, TCGA, gnomAD, and dbSNP, designed to annotate somatic variants and provide a classification according to ACMG guidelines (1). VCF files were generated using the default pipeline settings; unless on the cancer hotspot list (available upon request), splice site variants beyond ± 2 bp, intronic variants, and synonymous changes have been excluded from the analysis. CNVs were analysed using a custom in-house pipeline at RMH: CNVseeker v2.1. Quality metrics produced by the DRAGEN analysis are corrected for overall coverage before removing sequencing bias using a panel and gender-specific reference, then adjusted for GC content bias. This pipeline employs a circular binary segmentation algorithm to define larger chromosomal aberrations and generates noise scores based on normalised coverage data. For this study, the samples were screened only for missense, frameshift, in/del, or splicing-site variants in the *DDX41* gene. For patients in whom a somatic driver was detected in the apparent absence of a constitutional sequence variant, further analysis was undertaken to check for an unidentified germline copy number variant. We focused on samples with the well-known pathogenic somatic *DDX41* variant R525H, the most common somatic variant detected in samples with co-occurring germline variants in our study, as well as in other reports in the literature (2-4).

### Germline validation

Persistent variants with an allele frequency between 40% and 60% and no change in their variant allele frequency (VAF) following therapy or remission were deemed to be of germline origin. Variants in patients without a subsequent remission sample were considered to be of germline origin if the variant allele frequency (VAF) was between 40% and 60% and the tumour content was less than 20%. Confirmation of constitutional origin by analysis of DNA from cultured fibroblasts was undertaken, where clinically indicated (i.e. to inform onward management of the family), in a germline laboratory.

## RMH HaemOnc Full Panel Scope

Genes included in the RMH HaemOnc NGS panel are *ABCA1, ABL1, ACD, AKT1, ANKRD26, ARAF, ARID1A, ASXL1, ATM, ATR, ATRX, BCL11B, BCL2, BCL6, BCOR, BCORL1, BCR, BIRC3, BOD1L1, BRAF, BRCA1, BRCA2, BRCC3, BTK, CALR, CARD11, CBL, CBLB, CCND1, CCND2, CCND3, CD274, CD79B, CDKN2A, CDKN2B, CEBPA, CHEK2, CREBBP, CSF3R, CSNK1A1, CTC1, CTCF, CUX1, CXCR4, DCLRE1C,* ***DDX41(exon 1-17)****, DIS3, DKC1, DNAJC21, DNM2, DNMT3A, DNMT3B, DPYD, DUSP22, EED, ELANE, EP300, ERBB3, ETNK1, ETV6, EZH2, FAM46C, FANCL, FBXW7, FLT3, FOXO1, G6PC3, GATA1, GATA2, GFI1, GNAS, GNB1, GPRC5A, HAX1, HRAS, IDH1, IDH2, IKZF1, IL7R, IRF4, JAGN1, JAK1, JAK2, JAK3, KDM5A, KDM6A, KIT, KLF2, KMT2A, KMT2C, KMT2D, KMT2E, KRAS, LAMB4, LMO1, LMO2, MAP2K1, MAP3K1, MECOM, MEF2B, MET, MPL, MYB, MYC, MYCN, MYD88, NCOR1, NCOR2, NF1, NF2, NFE2, NOTCH1, NOTCH2, NPM1, NRAS, NRD1, NSD1, NT5C2, NTRK1, NTRK2, NTRK3, NUDT15, NUP98, PALB2, PAX5, PDGFRA, PDS5B, PHF6, PIGA, PIK3CA, PIK3CD, PIK3R1, PLCG2, PMS1, PMS2, PPM1D, PRPF8, PTEN, PTPN11, PTPRT, RAD21, RAD50, RAD51C, RAD51D, RHOA, RIT1, RPL5, RPL10, RUNX1, SAMD9, SAMD9L, SAMHD1, SBDS, SETBP1, SF1, SF3B1, SH2B3, SMC1A, SMC3, SRSF2, STAG2, STAT3, STAT5B, SUZ12, TAL1, TERC, TERT, TET1, TET2, TINF2, TP53, TPMT, TRAF2, U2AF1, U2AF2, USP7, VPS45, WAS, WT1, XPO1, XRCC2, ZRSR2*.

*DDX41*‐specific variant classification and interpretation

Due to the absence of gene-specific guidelines for *DDX41* variant interpretation, we applied the CanVIG-UK Consensus Specification for Cancer Susceptibility Genes and ACGS Best Practice Guidelines for Variant Classification (v3.0). Variants were classified as pathogenic (P), likely pathogenic (LP), or variant of uncertain significance (VUS) based on the cumulative quantity and strength of evidence supporting pathogenicity or benignity. The *DDX41* mane select reference transcript NM_016222.4 was utilised. Variant interpretation was independently performed by two clinical scientists.

Evidence criteria applied:

**PM2 (Population frequency):** Variants were queried in the Genome Aggregation Database (gnomAD) v4.1. PM2 was applied at moderate strength if the variant was absent from this database, or at supporting strength if the allele frequency was ≤0.002%.

**PVS1 (Loss-of-function variants):** Applied to variants predicted to cause loss of function. Eligible variant types included nonsense, frameshift, canonical splice site, and stop-loss variants. This code was applied on the basis that LOF seem to be a primary mechanism of disease for the *DDX41* mutation. Evidence strength was assigned following the decision tree described by Tayoun et al.(5)

**PP3 (*in silico* predictions):** Applied at supporting strength for missense variants with REVEL(6) scores >0.7 or for variants at intron-exon boundaries with SpliceAI (7) Δ scores ≥0.2, indicating potential deleterious impact.

**PS4 (Case-control data):** Due to the limited availability of large-scale case-control datasets for *DDX41*, formal case-control analyses were not feasible. Instead, a case-counting approach was applied. ClinVar was used to identify previously reported cases in which the variant had been detected in affected individuals, and the published literature was reviewed for additional reports. PS4 was applied at a maximum strength of supporting evidence under two circumstances; 1)where PM2 was applicable, supporting PS4 was assigned if the variant had been reported in multiple unrelated individuals with myeloid neoplasms, 2)where PM2 was not applicable (e.g., variants observed at very low frequency in population databases consistent with late-onset disease), PS4 was still considered at a supporting level if multiple independent reports were available in the literature, consistent with CanVIG recommendations for more common or later-onset predisposition syndromes. Given the current lack of robust case-control data for *DDX41*, the application of PS4 was deliberately conservative, restricted to supporting strength only.

**PM5 (Missense variants at the same residue):** This was applied at moderate strength when a different missense variant at the same amino acid residue was documented in ClinVar or the published literature as pathogenic or likely pathogenic in individuals with myeloid neoplasms.

**PS3 (Functional studies):** This was applied at supporting strength where published functional or biochemical studies demonstrated a damaging effect on DDX41 protein function relevant to disease pathogenesis. Eligible evidence included studies examining protein-protein interactions, RNA binding capacity, helicase activity, or effects on downstream transcriptional targets. Due to the limited availability of functional data for most *DDX41* variants at present, this criterion was applied only where robust experimental evidence supported pathogenicity.

1. Li MM, Datto M, Duncavage EJ, Kulkarni S, Lindeman NI, Roy S, et al. Standards and Guidelines for the Interpretation and Reporting of Sequence Variants in Cancer: A Joint Consensus Recommendation of the Association for Molecular Pathology, American Society of Clinical Oncology, and College of American Pathologists. J Mol Diagn. 2017;19(1):4-23.

2. Polprasert C, Schulze I, Sekeres MA, Makishima H, Przychodzen B, Hosono N, et al. Inherited and Somatic Defects in DDX41 in Myeloid Neoplasms. Cancer Cell. 2015;27(5):658-70.

3. Sebert M, Passet M, Raimbault A, Rahme R, Raffoux E, Sicre de Fontbrune F, et al. Germline DDX41 mutations define a significant entity within adult MDS/AML patients. Blood. 2019;134(17):1441-4.

4. Lewinsohn M, Brown AL, Weinel LM, Phung C, Rafidi G, Lee MK, et al. Novel germ line DDX41 mutations define families with a lower age of MDS/AML onset and lymphoid malignancies. Blood. 2016;127(8):1017-23.

5. Abou Tayoun AN, Pesaran T, DiStefano MT, Oza A, Rehm HL, Biesecker LG, et al. Recommendations for interpreting the loss of function PVS1 ACMG/AMP variant criterion. Hum Mutat. 2018;39(11):1517-24.

6. Ioannidis NM, Rothstein JH, Pejaver V, Middha S, McDonnell SK, Baheti S, et al. REVEL: An Ensemble Method for Predicting the Pathogenicity of Rare Missense Variants. Am J Hum Genet. 2016;99(4):877-85.

7. de Sainte Agathe JM, Filser M, Isidor B, Besnard T, Gueguen P, Perrin A, et al. SpliceAI-visual: a free online tool to improve SpliceAI splicing variant interpretation. Hum Genomics. 2023;17(1):7.
